# Pro-inflammatory role of Wnt/β-catenin signaling in endothelial dysfunction

**DOI:** 10.3389/fcvm.2022.1059124

**Published:** 2023-01-17

**Authors:** Kerry S. Wadey, Alexandros Somos, Genevieve Leyden, Hazel Blythe, Jeremy Chan, Lawrence Hutchinson, Alastair Poole, Aleksandra Frankow, Jason L. Johnson, Sarah J. George

**Affiliations:** ^1^Bristol Medical School, Translational Health Sciences, University of Bristol, Bristol, United Kingdom; ^2^School of Physiology, Pharmacology and Neuroscience, Translational Health Sciences, University of Bristol, Bristol, United Kingdom

**Keywords:** Wnt/β-catenin, TNF-α, NFκB, endothelium, monocyte, permeability, platelet, atherosclerosis

## Abstract

**Background:**

Endothelial dysfunction is a critical component of both atherosclerotic plaque formation and saphenous vein graft failure. Crosstalk between the pro-inflammatory TNF-α-NFκB signaling axis and the canonical Wnt/β-catenin signaling pathway potentially plays an important role in regulating endothelial dysfunction, though the exact nature of this is not defined.

**Results:**

In this study, cultured endothelial cells were challenged with TNF-α and the potential of a Wnt/β-catenin signaling inhibitor, iCRT-14, in reversing the adverse effects of TNF-α on endothelial physiology was evaluated. Treatment with iCRT-14 lowered nuclear and total NFκB protein levels, as well as expression of NFκB target genes, IL-8 and MCP-1. Inhibition of β-catenin activity with iCRT-14 suppressed TNF-α-induced monocyte adhesion and decreased VCAM-1 protein levels. Treatment with iCRT-14 also restored endothelial barrier function and increased levels of ZO-1 and focal adhesion-associated phospho-paxillin (Tyr118). Interestingly, inhibition of β-catenin with iCRT-14 enhanced platelet adhesion in cultured TNF-α-stimulated endothelial cells and in an *ex vivo* human saphenous vein model, most likely *via* elevating levels of membrane-tethered vWF. Wound healing was moderately retarded by iCRT-14; hence, inhibition of Wnt/β-catenin signaling may interfere with re-endothelialisation in grafted saphenous vein conduits.

**Conclusion:**

Inhibition of the Wnt/β-catenin signaling pathway with iCRT-14 significantly recovered normal endothelial function by decreasing inflammatory cytokine production, monocyte adhesion and endothelial permeability. However, treatment of cultured endothelial cells with iCRT-14 also exerted a pro-coagulatory and moderate anti-wound healing effect: these factors may affect the suitability of Wnt/β-catenin inhibition as a therapy for atherosclerosis and vein graft failure.

## Introduction

The endothelium bears enormous physiological importance by serving to modulate inflammatory processes, selective passage of materials across the vessel wall, vascular tone and blood viscosity. Detrimental shifts in endothelial behavior are collectively referred to as “endothelial dysfunction” and are tightly correlated with early-and late-stage atherosclerosis and saphenous vein graft degeneration (1, 2). Such phenotypic shifts include elevated levels of secreted inflammatory cytokines, compromised barrier function, an upregulation of adhesion molecules, and reduced anti-coagulatory potential.

Current research stresses the significance of endothelial dysfunction in the pathogenesis and progression of atherosclerosis. Increased endothelial permeability at lesion-prone sites in the arterial vasculature permits infiltration of circulating low density lipoprotein (LDL) (3), setting in motion a cascade of pathogenic events. Presentation of adhesion molecules on the endothelial surface promotes monocyte attachment and recruitment (4–6). Endothelial-and macrophage-derived pro-inflammatory signals also stimulate medial vascular smooth muscle cell (VSMC) intimal migration and proliferation, culminating in intimal hyperplasia (5). Hence, endothelial dysfunction is an essential component in the initiation and development of atherosclerotic lesions.

Saphenous vein graft degeneration is also a direct consequence of endothelial dysfunction. During coronary artery bypass graft (CABG) surgery, transient hypoxia (7), high pressure distension (8), and handling of the vein conduit during harvest and implantation (9) damages the endothelium and exacerbates dysfunction. Endothelial denudation accounts for thrombotic occlusion typically within the one month of surgery in 3–12% of patients (10, 11). Non-occlusive thrombi may also become progressively organized into vascular tissue, in which platelets release cytokines and growth factors that compound inflammation, VSMC proliferation and neointima formation (12). Thus, in designing novel therapeutic interventions for endothelial dysfunction in vein graft failure, one must also consider whether the approach would impair re-endothelialisation and elevate risk of thrombus formation.

**FIGURE 1:**
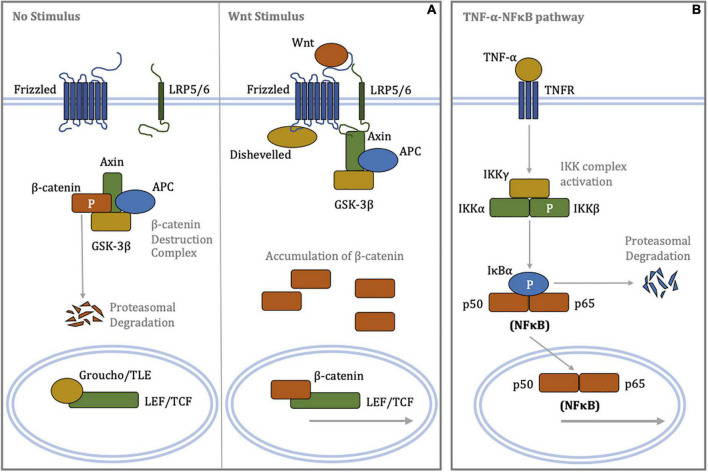
Schematic diagrams of signal transduction in the canonical Wnt/β-catenin and canonical TNF-α-NFκB signaling pathways. **(A)** (Left panel) In the absence of a Wnt stimulus, β-catenin is phosphorylated by the destruction complex targeting it for poly-ubiquitination and subsequent proteasome-mediated proteolysis. Low cytoplasmic levels of β-catenin permit repression of LEF/TCF-dependent transcription by Groucho/TLE. (Right panel) Binding of Wnt ligands to the Frizzled/LRP5/6 receptor complex promotes recruitment of Disheveled and Axin, resulting in disassembly of the β-catenin destruction complex. Cytoplasmic accumulation of β-catenin allows for its nuclear translocation, where β-catenin displaces Groucho/TLE from LEF/TCF, enabling transcriptional regulation. **(B)** Binding of the TNF-α ligand to the TNF receptor recruits, phosphorylates and activates the IKK complex *via* oligomerisation of a series of adaptor proteins. In turn, the activated IKK complex phosphorylates IκBα, resulting in poly-ubiquitination of IκBα and its rapid proteasomal degradation. The unbound NFκB heterodimer complex, where p50 and p65 are members of NFκB family, translocates to the nucleus and regulates transcription of target genes involved inflammation. APC, adenomatous polyposis coli; GSK-3β, glycogen synthase kinase-3β; IκBα, inhibitor of κB-α; IKK, IκB kinase; LEF, lymphoid enhancer-building factor; LRP5/6, lipoprotein receptor-related protein 5/6; NFκB, nuclear factor kappa-light-chain-enhancer of activated B cells; P, phosphorylation; TCF, T-cell factor; TLE, transducin-like enhancer of split; TNF-α, tumor necrosis factor-α; TNFR, tumor necrosis factor receptor.

The TNF-α-NFκB signaling axis is a highly potent pro-inflammatory pathway and mediates multiple aspects of endothelial dysfunction. Reciprocal cross-regulation between the canonical Wnt/β-catenin and NFκB signaling pathways is complex as there are multiple regulatory mechanisms, the nature of which can be either repressive or enhancing (see Figure 1 for mechanisms of signal transduction) (13). Crosstalk as such enables individual pathways to broaden their range of biological function (13); in turn, this implies that pharmacological inhibitors targeting any one pathway may also be able to extend their therapeutic effect. In this study, we demonstrate the capacity of a Wnt/β-catenin signaling inhibitor, iCRT-14, to repress NFκB activation as well as partially recover a non-inflammatory, functional state in endothelial cells of arterial and venous origin.

## Materials and methods

### Culture of HUVECs and HCAECs

Human umbilical vein endothelial cells (HUVECs, pooled from up to four different donors per lot) and human coronary artery endothelial cells (HCAECs, single donor per lot) from different donors were purchased from Promocell (C-12203 and C-12221), and were utilized between passages 2 and 6. Experiments were repeated with endothelial cells from different lots. HUVECs and HCAECs were cultured in endothelial cell growth medium (Promocell; C-22010 and C-22020) or 2% (v/v) FBS endothelial cell basal medium (Promocell; C-22210 and C-22220) supplemented with 100 mg/mL penicillin and 100 IU/mL streptomycin. Human recombinant TNF-α protein was procured from R&D Systems (210-TA-20). Pharmacological inhibitor of β-catenin, iCRT-14, was acquired from Tocris Bioscience (4299). Cells were maintained in a humified atmosphere with 5% CO_2_ at 37°C.

### Culture of THP-1 cells

THP-1 cells purchased from ATCC (TIB-202) were maintained in Roswell Park Memorial Institute-1640 (RPMI-1640: Gibco; 31870-025) culture medium supplemented with 10% (v/v) FBS, 2 mM L-glutamine, 100 mg/mL penicillin and 100 IU/mL streptomycin. Cells were cultured in a humified atmosphere with 5% CO_2_ at 37°C.

### Adhesion assay

Human umbilical vein endothelial cells (HUVECs) or HCAECs were grown to confluency; following treatment, endothelial cells were co-cultured with calcein-AM (10 μM)-labeled THP-1 cells at a concentration of 1 × 10^4^ cells/mL for 30 min. Non-adherent THP-1 cells were removed by gentle washing with PBS, and adherent THP-1 cells were visualized under a fluorescent live cell imaging microscope.

### Permeability assay

Human umbilical vein endothelial cells (HUVECs) or HCAECs were seeded into sterile 6.5 mm Transwell inserts with a 0.4 μm pore polyester membrane (Corning; 3470) and cultured to confluency. A confluent endothelial monolayer was determined by transferring Transwell inserts to empty wells and refreshing cell culture medium in the top chamber with 500 μl endothelial cell growth medium; when media no longer permeated through to the lower chamber within 5 min, the endothelium was deemed intact. Post-treatment, 60 ng/mL streptavidin-HRP (R&D Systems; DY998) diluted in serum-free endothelial cell basal medium was added to the top chamber and the diffusion rate to the bottom chamber was assessed after 5 min. For detection of streptavidin-HRP, 20 μL cell culture medium was retrieved from the bottom chamber, to which 50 μL Substrate Reagent (R&D Systems; DY999) was added for 5 min. Subsequently, 25 μL Stop Solution 2N Sulfuric Acid (R&D Systems; DY994) was added. Spectrum absorption was measured at 450 nm using an ELISA plate reader.

### Proliferation assay

Culture medium was supplemented with 10 μM EdU and incorporation quantified *via* fluorescently labeling using the EdU-Click 488 Imaging Kit (Sigma-Aldrich; BCK-EDU488).

### Scratch wound assay

Human umbilical vein endothelial cells (HUVECs) or HCAECs were grown to confluency, scratched with a 1 mL pipette tip to draw an X-shaped wound, and then culture medium replaced and supplemented with treatments. Wound healing was allowed for 18 h and migrated distance measured.

### Washed platelet preparation

Human blood was collected from healthy volunteers (Ethics number: REC 10/H0107/32) in 4.5 mL BD Vacutainer Citrate Tubes (Fisher Scientific; BD367691). Platelet-rich plasma (PRP) was obtained by centrifugation at 180 *g* for 17 min. PRP was isolated then supplemented with 0.02 U/mL apyrase, 140 nM prostaglandin E1 (PGE1; Sigma-Aldrich; P7527), and a 6:1 ratio of PRP to ACD (85 mM trisodium citrate, 71 mM citric acid, 111 mM dextrose). Platelet pellets were obtained by centrifugation at 520 *g* for 10 min and washed in CGS buffer (120 mM NaCl, 12.9 mM trisodium citrate, 30 mM dextrose, pH 6.5) supplemented with 0.02 U/mL apyrase and 140 nM prostaglandin E1. Platelets were centrifuged at 520 *g* for 10 min, resuspended in Tyrode’s buffer [10 mM HEPES, 145 mM NaCl, 3 mM KCl, 0.5 mM NaH_2_PO_4_, 1 mM MgSO_4_, 0.1% (w/v) dextrose, pH 7.2] supplemented with 0.02 U/mL apyrase, 140 nM prostaglandin E1 and 4 μg/mL BCECF-AM (Sigma-Aldrich; B8806) and incubated for 30 min at room temperature. Platelets were centrifuged at 520 *g* for 10 min, adjusted to a concentration of 8 × 10^7^ platelets/mL in Tyrode’s buffer supplemented with 2 mM CaCl_2_, 1 mM MgCl_2_, and 0.5 U/mL bovine thrombin (Sigma-Aldrich; T4648), and incubated for 10 min at room temperature. To inactivate thrombin, platelets were then incubated with 2 U/mL hirudin for a further 10 min at room temperature.

### *In vitro* platelet adhesion assay

Human umbilical vein endothelial cells (HUVECs) were grown to confluency in 24-well culture plates; following treatment, endothelial cells were co-cultured with 500 μL of activated and BCECF-AM-labeled platelets for 10 min at 37°C, 5% CO_2_ in a humidified atmosphere. Non-adherent platelets were removed by gently washing twice with Tyrode’s buffer supplemented with 2 mM CaCl_2_ and 1 mM MgCl_2_. Adherent platelets and HUVECs were then lysed in 100 μL of lysis buffer [0.1% (w/v) SDS, 30 mM Tris, pH 8.8] and the fluorescence signal of BCECF-AM measured using a plate reader with excitation and emissions wavelengths of 485 and 535 nm, respectively.

### *Ex vivo* platelet adhesion assay

Surplus surgically prepared human saphenous vein was collected from consenting patients (Ethics number: REC14/EE/109). Segments of vein were dissected in endothelial cell growth medium: veins were opened along their longitudinal axis and 5–10 mm transverse segments cut. In Sylgard resin-coated petri dishes, the four corners of the saphenous vein segments were pinned down onto mesh using minute pins and cultured in 5 mL of endothelial cell growth medium for 24 h at 37°C, 5% CO_2_ in a humidified atmosphere. Vein segments were then co-cultured with 5 mL of activated and BCECF-AM-labeled platelets (prepared as described in Section “2.7 Washed platelet preparation”) for 10 min at 37°C, 5% CO_2_ in a humidified atmosphere. Non-adherent platelets were removed by gently washing twice with Tyrode’s buffer supplemented with 2 mM CaCl_2_ and 1 mM MgCl_2_. Vein segments were coated with Immersion medium Immersol W 2010 (Zeiss; 444969), mounted onto glass slides with the luminal side facing downward then imaged immediately on the Zeiss AxioObserver Z1 fluorescent miscroscope. Z-stack images were taken of 6 randomly selected fields. Adherent platelets were counted manually.

### Immunofluorescence

Cells were fixed with 3% (w/v) paraformaldehyde/PBS for 10 min, then permeabilised with 0.1–1.0% (v/v) Triton X-100/PBS for 15 min. To localize membrane-tethered vWF (excluding intracellular vWF), this permeabilisation step was not performed. Cells were subsequently blocked with 20% (v/v) goat serum/PBS for 30 min, then incubated with primary antibody diluted in PBS, overnight at 4°C. Primary antibodies included: anti-cleaved caspase-3 IgG (R&D Systems; MAB835), anti-NFκB p65 IgG (Cell Signaling Technology; 8242), anti-phospho-paxillin (Tyr118) IgG (Invitrogen; 44-722G), anti-VE-Cadherin IgG (Cell Signaling Technology; 2500), anti-von Willebrand factor IgG (Cell Signaling; 65707S), and anti-ZO-1 IgG (Abcam; ab221547). Cells were then incubated with biotinylated secondary antibody diluted 1:200 in PBS for 30 min, followed by Dylight 488 Streptavidin (Vector Laboratories; SA-5488-1) diluted 1:200 in PBS for 30 min. Prolong Gold Antifade Reagent with DAPI (ThermoFisher Scientific; P36935) was used for staining nuclei and mounting.

### ELISA

The concentration of secreted MCP-1, IL-6, and IL-8 in cell culture supernates was evaluated using Quantikine ELISA Kits (R&D Systems; DCP00, D6050, D8000C), in accordance with manufacturer’s instructions.

### Concentration of conditioned culture medium

Cell culture conditioned media were collected from endothelial cells 18 h post-treatment and concentrated using Amicon Ultra-0.5 Centrifugal Filter Units (Millipore; UFC5010), in accordance with manufacturer’s instructions. For 500 μL of conditioned culture medium, the final concentrate volume was 22 μL and the concentration factor 22×.

### Western blotting

Cells were homogenized in SDS lysis buffer [1% (w/v) SDS, 50 mM Tris–HCl (pH 8), 10% (v/v) glycerol] before mechanical disruption with the insert of a 1 mL syringe. Protein concentrations were calculated using the Micro Bicinchoninic Acid (BCA) Assay Kit (ThermoFisher Scientific; 23235), in accordance with manufacturer’s instructions; protein concentration was then normalized across samples by dilution with HPLC water. One volume Laemmli Sample Buffer (Bio-Rad; 161-0737) supplemented with 5% (v/v) β-mercaptoethanol was added to 10–20 μL of lysate before heating to 95°C for 5 min. For secretory proteins, one volume Laemmli Sample Buffer supplemented with 5% (v/v) β-mercaptoethanol was added to 20 μL of concentrated conditioned culture medium (prepared as described in Section “2.12 Concentration of conditioned culture medium”) before heating to 95°C for 5 min. Prepared samples and BLUeye Prestained Protein Ladder (Geneflow; S6-0024) were loaded onto Mini-PROTEAN TGX Stain-Free Protein Gels (Bio-Rad; 4568084) and electrophoresed in Tris/Glycine/SDS running buffer (Bio-Rad; 161-0772) at 300 V for 20 min. Protein was normalized again with in-gel stain-free bands visualized *via* a 1-min exposure to UV light. Subsequently, protein was transferred to Trans-Blot Nitrocellulose Membranes (Bio-Rad; 170-4158) using the Trans-Blot Turbo Transfer Starter System (Bio-Rad; 170-4155).

Nitrocellulose membranes were blocked with either 5% (w/v) BSA or 5% (w/v) fat-free milk powder/TBST [20 mM Tris, 137 mM NaCl, 0.1% (v/v) Tween 20; pH 7.6] for 30 min, followed by an overnight incubation at 4°C with primary antibody diluted in 5% (w/v) BSA/TBST. Membranes were washed with TBST, incubated with HRP-conjugated secondary antibody for 1 h, and washed again with TBST. Luminata Forte Western HRP substrate (Merck Millipore; WBLUF0100) was utilized for signal detection. Primary antibodies included anti-ADAMTS13 IgG (Abcam; ab177940), anti-ICAM-1 IgG (Abcam; ab53013), anti-IκBα IgG (Cell Signaling Technology; 4814), anti-integrin α_*v*_ IgG (Cell Signaling Technology; 4711), anti-integrin β_3_ IgG (Cell Signaling Technology; 13166), anti-NFκB p65 IgG (Cell Signaling Technology; 8242), anti-VCAM-1 IgG (Abcam; ab134047), and anti-von Willebrand factor IgG (Cell Signaling; 65707S).

### Statistics

For two groups, data were analyzed by Student *t*-tests. For three or more groups, data were analyzed by ANOVA and Student-Newman-Keuls Multiple Comparisons *post-hoc* test. Experiments were repeated 3 to 8 times with cells or tissue from different donors. Findings were considered statistically significant when *p* < 0.05.

## Results

### Inhibition of Wnt/β-catenin signaling disrupted the TNF-α-NFκB pathway

Primary cultures of HUVECs were challenged with 10 ng/mL recombinant human TNF-α in 2% (v/v) FBS basal culture medium for 18 h. Endothelial cells were simultaneously treated with either 25 μM of inhibitor of β-catenin-responsive transcription-14 (iCRT-14) or 0.05% DMSO vehicle control. Cells not stimulated with TNF-α served as a negative control. The small molecule compound iCRT-14 has been reported to display low promiscuity and to potently inhibit the canonical Wnt/β-catenin signaling pathway (IC_50_ = 40.3 nM) (14, 15). Gonsalves and colleagues showed that iCRT-14 suppresses this pathway by most likely blocking the interface *via* which β-catenin and T-cell factor 4 (TCF4) interact, thereby leading to impairment of transcriptional regulation of downstream genes (14, 15). Additionally, the authors demonstrated that, at a dose of 25 μM, iCRT-14 does not indiscriminately interfere with other protein partners of β-catenin such as E-cadherin and α-catenin, thereby conserving the function of β-catenin in stabilizing adherens junctions, and that it does not modulate β-catenin-independent non-canonical Wnt signaling. Gonsalves et al. also established that in addition to inhibiting β-catenin-TCF4 protein-protein interaction, iCRT-14 may also influence binding of TCF/LEF transcription factors to their consensus DNA binding sites, providing a further mechanism for suppression of β-catenin and TCF-mediated signaling (14).

To validate inhibition of canonical Wnt signaling by iCRT-14, expression of the β-catenin-responsive gene axin-2 was quantified by Western blotting. In TNF-α-stimulated HUVECs, treatment with 25 μM iCRT-14 significantly suppressed protein levels of axin-2 compared to cells challenged with TNF-α alone, demonstrating effective inhibition of β-catenin-TCF4 transcriptional activity (TNF-α: 1.16 ± 0.04; TNF-α + iCRT-14: 0.78 ± 0.07, fold change from control, *p* < 0.05, *n* = 8).

**FIGURE 2:**
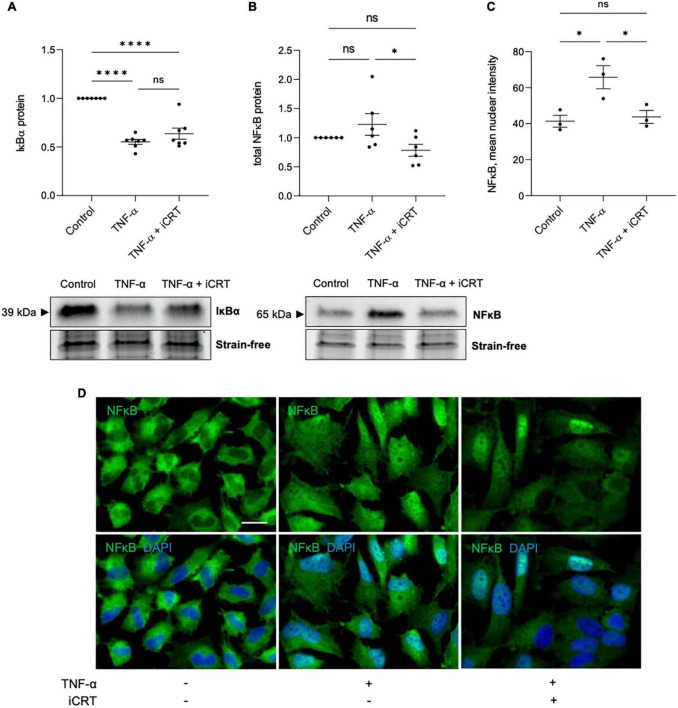
Inhibition of Wnt/β-catenin signaling disrupted the inflammatory TNF-α-NFκB signaling axis. HUVECs were stimulated with 10ng/mL recombinant human TNF-α in the presence of either 0.05% DMSO vehicle control or 25μM iCRT for 18 h. **(A)** IκBα from whole cell lysates was quantified by Western blotting, normalized to stain-free loading controls and expressed as a fold change of control (*n* = 7). Representative Western blot and stain-free loading control shown. **(B)** NFκB from whole cell lysates was quantified by Western blotting, normalized to stain-free loading controls and expressed as a fold change of control (*n* = 6). Representative Western blot and stain-free loading control shown. **(C)** Following immunofluorescence for NFκB, mean nuclear NFκB levels were quantified using a Fiji-based macro (*n* = 3). **(D)** Representative images of HUVECs immunostained for NFκB (green). Nuclei were stained with DAPI (blue). Scale bar represents 10μm and applies to all panels. *p < 0.05, **p < 0.01, ***p < 0.001, and ****p < 0.0001. ns, not significant.

Transduction of TNF-α-NFκB signaling was assessed by using Western blotting to quantify IκBα and NFκB protein levels, and immunofluorescence to monitor subcellular localization of NFκB. As anticipated, activation of HUVECs with TNF-α reduced IκBα protein expression compared to control group; addition of 25 μM iCRT-14 did not restore or otherwise significantly influence IκBα levels (Figure 2A). TNF-α promoted nuclear localization of NFκB as expected (Figures 2C, D); additional treatment with iCRT-14 lowered levels of nuclear NFκB with respect to HUVECs treated with TNF-α alone (Figures 2C, D). Western blotting with whole cell lysates showed that TNF-α did not consistently or significantly increase total NFκB protein levels; interestingly, addition of iCRT-14 significantly suppressed total NFκB levels compared to cells treated with TNF-α alone (Figure 2B). These findings suggest that canonical Wnt/β-catenin signaling facilitates the inflammatory NFκB pathway in TNF-α-activated endothelial cells.

NFκB is a central mediator of pro-inflammatory cytokine production. ELISA immunoassays showed a surge in release of MCP-1, IL-6, and IL-8 cytokines in response to challenge of HUVECs and HCAECs with TNF-α. Treatment with iCRT-14 significantly reversed levels of secreted MCP-1 and IL-8, but not IL-6 in both cell types (Table 1).

**TABLE 1:** Inhibition of Wnt/β-catenin suppressed TNF-α-induced MCP-1 and IL-8 secretion, but not IL-6.

Inflammatory cytokine	HUVECs/%	HCAECs/%
MCP-1	-42.8 ± 8.2*	-35.7 ± 12.6*
IL-6	+ 127 ± 55.3	-5.32 ± 17.8
IL-8	-31.6 ± 7.77*	-44.7 ± 9.94*

HUVECs and HCAECs were stimulated with 10 ng/mL recombinant human TNF-α in the presence of either 0.05% DMSO vehicle control or 25 μM iCRT for 18 h. Secreted inflammatory cytokines were quantified by ELISA, and results expressed as TNF-α + iCRT as a percentage difference from TNF-α (*n* = 4–6). *Indicates *p* < 0.05.

### Inhibition of Wnt/β-catenin signaling lowered leukocyte adhesion

**FIGURE 3:**
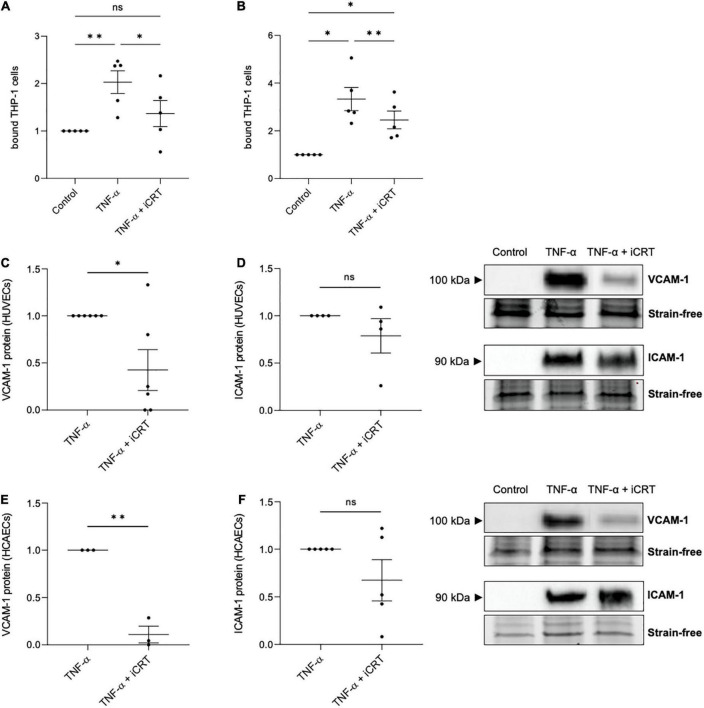
Inhibition of Wnt/β-catenin signaling reduced TNF-α-induced monocyte-adhesion. Cultured endothelial cells were stimulated with 10 ng/mL recombinant human tumor necrosis factor-α (TNF-α) and supplemented with either 0.05% DMSO vehicle control or 25 μM inhibitor of β-catenin-responsive transcription (iCRT) for 18 h. Calcein-labeled THP-1 cells were allowed to adhere to human umbilical vein endothelial cells (HUVECs) **(A)** or HCAECs **(B)** for 30 min, then adherent cells were quantified and expressed as a fold change of control (*n* = 5 each). In HUVECs, VCAM-1 **(C)**, and ICAM-1 **(D)** from whole cell lysates were quantified by Western blotting, normalized to stain-free loading controls and expressed as a fold change of TNF-α (*n* = 6 and 4, respectively). Representative Western blots shown. In HCAECs, VCAM-1 **(E)**, and ICAM-1 **(F)** from whole cell lysates were quantified by Western blotting, normalized to stain-free loading controls and expressed as a fold change of TNF-α (*n* = 3 and 5, respectively). Representative Western blots and stain-free loading controls are shown. *Indicates *p* < 0.05, ^**^p < 0.01, ^***^p < 0.001, and ^****^p < 0.0001. NS denotes not significant.

Confluent HUVECs and HCAECs were unchallenged or stimulated with 10 ng/mL recombinant human TNF-α for 18 h, in the presence of 0.05% DMSO vehicle control or 25 μM iCRT-14. Endothelial cells were then co-cultured with calcein-AM-labeled THP-1 cells, a human monocytic cell line, and adherent cells quantified. In unchallenged HUVECs, treatment with iCRT-14 did not influence endothelial-THP-1 cell interaction (Supplementary Figure 1A). Consistent with current literature, TNF-α produced a notable increase in the number of adherent monocytic cells (Figures 3A, B). This was significantly dampened by addition of iCRT-14, demonstrating the potential anti-inflammatory benefits of pharmacologically suppressing canonical Wnt/β-catenin signaling (Figures 3A, B).

TNF-α-induced transcriptional up-regulation of vascular cell adhesion molecule-1 (VCAM-1) and intercellular adhesion molecule-1 (ICAM-1) *via* NFκB activation is well-documented (16). Concordantly, in both HUVECs and HCAECs, Western blotting confirmed a marked induction in VCAM-1 and ICAM-1 following TNF-α stimulation, where no protein was detected in unstimulated cells (Figures 3C–F). Treatment with iCRT-14 significantly reversed the elevation in VCAM-1 expression (Figures 3C, E), though did not consistently lower ICAM-1 levels (Figures 3D, F). These data propose VCAM-1 depletion as one such mechanism by which the compound iCRT-14 impeded firm adhesion of THP-1 cells to the activated endothelial cells.

### Inhibition of Wnt/β-catenin signaling restored endothelial barrier function

**FIGURE 4:**
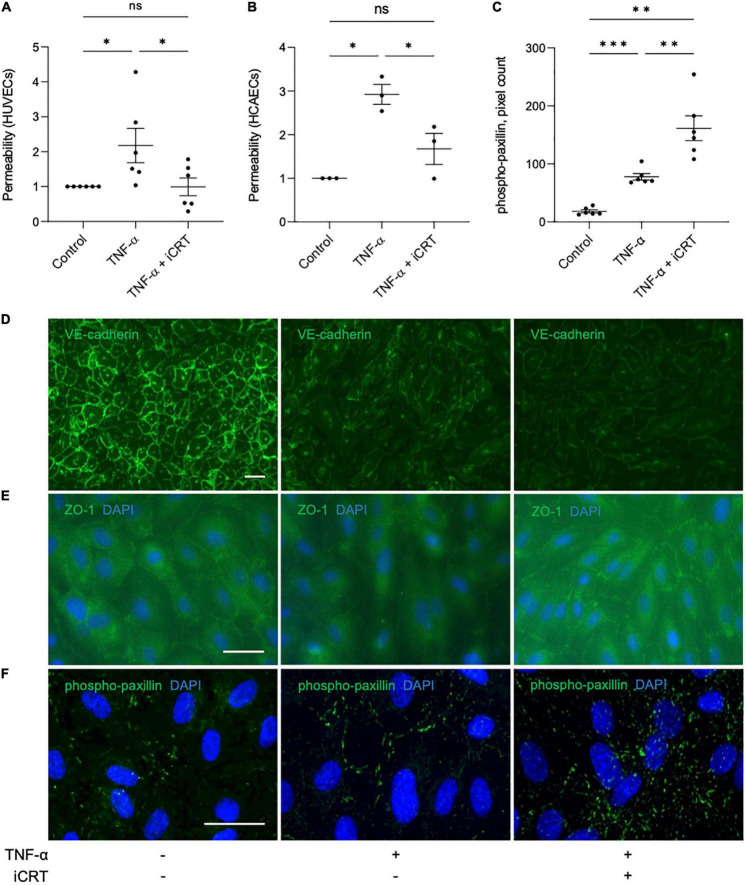
Inhibition of Wnt/β-catenin signaling restored barrier function in TNF-α-stimulated endothelial cells. Cultured endothelial cells were stimulated with 10 ng/mL recombinant human TNF-α in the presence of either 0.05% DMSO vehicle control or 25 μM inhibitor of β-catenin-responsive transcription (iCRT) for 18 h. Human umbilical vein endothelial cells (HUVECs) **(A)** or HCAECs **(B)** were seeded in Transwell inserts and streptavidin-HRP leakage across the endothelial monolayers was quantified. Data are expressed as a fold change of control (*n* = 6 and 3, respectively) **(C)**. In HUVECs, following immunofluorescence for phospho-paxillin (Tyr118), total phospho-paxillin (Tyr118) levels were quantified using a Fiji-based macro, and normalized to cell count (*n* = 6). Representative images of HUVECs immunostained (green) for VE-cadherin **(D)**, ZO-1 **(E)** or phospho-paxillin (Tyr118) **(F)**. Nuclei were stained with DAPI (blue). Scale bars represent 10 μm and apply to all panels. *Indicates *p* < 0.05, ^**^p < 0.01, ^***^p < 0.001, and ^****^p < 0.0001. NS denotes not significant.

TNF-α-NFκB signaling is a known contributor of increased endothelial permeability (17–19). To evaluate the potential of iCRT-14 in restoring endothelial barrier function, HUVECs and HCAECs were unchallenged or stimulated with 10 ng/mL recombinant human TNF-α for 18 h, and concurrently treated with either 0.05% DMSO vehicle control or 25 μM iCRT-14; endothelial permeability was then assessed by quantifying streptavidin-HRP leakage across the monolayer. In HUVECs unchallenged by TNF-α, addition of iCRT-14 did not affect endothelial permeability (Supplementary Figure 1B). As expected, TNF-α disrupted the intact HUVEC and HCAEC monolayers, and treatment with iCRT-14 successfully prevented this disruption to barrier function demonstrating its capacity to reduce vascular leakage (Figures 4A, B).

Vascular endothelial (VE)-cadherin is the cornerstone of endothelial barrier maintenance (20, 21). In this study, immunofluorescence confirmed an abundance of VE-cadherin protein at the cell-cell junctions in HUVECs (Figure 4D). A dramatic reduction in membrane-localized VE-cadherin following stimulation of cells with TNF-α was observed (Figure 4D), consistent with previous observations (22, 23). Curiously, despite improving barrier function, addition of iCRT-14 did not salvage levels of membrane-localized VE-cadherin (Figure 4D).

Zonula occludens (ZO) proteins are essential for the assembly and organization of tight junction complexes (17, 24, 25). In HUVECs, TNF-α-stimulation lowered levels of ZO-1 localized to the cell membrane (Figure 4E). Treatment with iCRT-14 restored levels of membrane-associated ZO-1 (Figure 4E).

Paxillin is a focal adhesion-associated adaptor protein that is phosphorylated at multiple residues, including Tyr118, upon engagement of integrins with extracellular matrix (26). Stimulation of HUVECs with TNF-α significantly increased the number of active focal adhesions, as indicated by immunofluorescence for phospho-paxillin (Tyr118); this increase was further enhanced by addition of iCRT-14 (Figures 4C, F).

### Inhibition of Wnt/β-catenin signaling increased platelet adhesion

**FIGURE 5:**
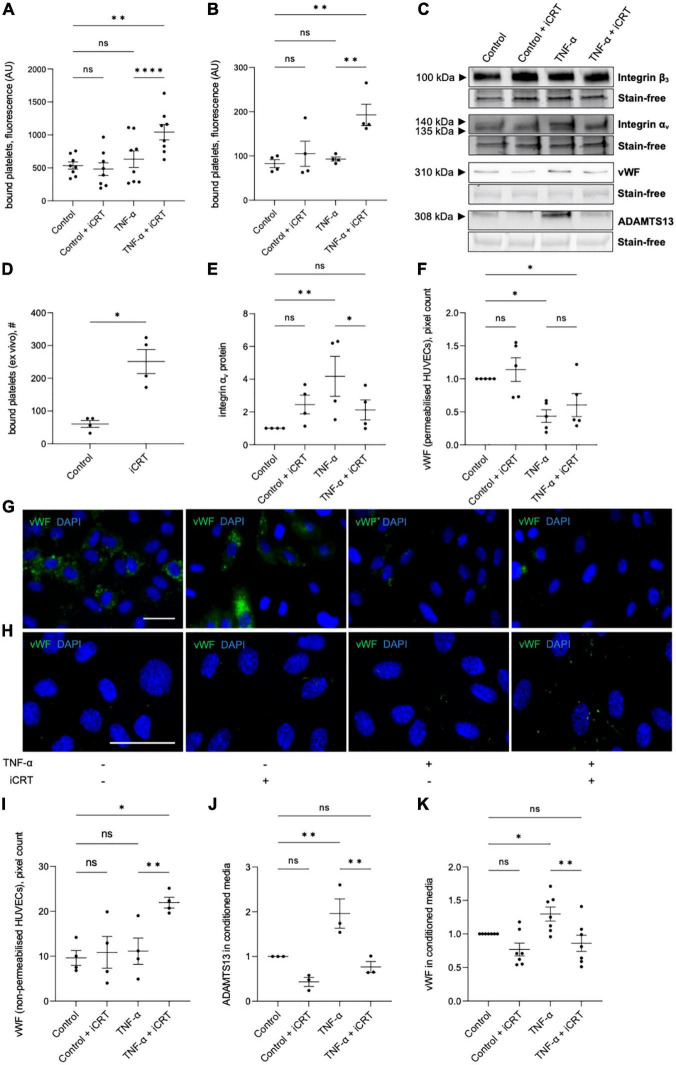
Inhibition of Wnt/β-catenin signaling enhanced platelet binding to TNF-α-stimulated endothelial cells. Cells were treated with either 0.05% DMSO vehicle control or 25 μM inhibitor of β-catenin-responsive transcription (iCRT) in the presence or absence of 10 ng/mL recombinant human TNF-α stimulus for 18 h. BCECF-AM-labeled, thrombin-activated platelets were co-cultured with human umbilical vein endothelial cells (HUVECs) **(A)** and HCAECs **(B)** for 10 min, bound platelets lysed, and the fluorescent signal quantified. Data are expressed as optical density (*n* = 8 and 4, respectively). **(C)** Representative Western blots for integrins α_v_ and β_3_ in whole cell lysates, and vWF and ADAMTS13 in conditioned media, and stain-free controls in HUVECs. **(D)** Segments of human saphenous vein were co-cultured with BCECF-AM-labeled, thrombin-activated platelets for 10 min, and the number of bound platelets quantified (*n* = 4). **(E)** In HUVECs, integrin α_v_ from whole cell lysates was quantified by Western blotting, normalized to stain-free controls and expressed as a fold change from control (*n* = 4). Quantification and representative images of immunofluorescence (green) for vWF in permeabilised **(F,G)** and non-permeabilised **(H,I)** HUVECs, to detect intracellular and membrane-tethered vWF, respectively (*n* = 5 and 4, respectively). Nuclei were stained with DAPI (blue). Scale bars represent 10 μm and apply to all panels. From cultured HUVECs, ADAMTS13 **(J)**, and soluble vWF **(K)** in conditioned culture medium were quantified by Western blotting, normalized to stain-free controls and expressed as a fold change from control (*n* = 7 and 3, respectively). *Indicates *p* < 0.05, ^**^p < 0.01, ^***^p < 0.001, and ^****^p < 0.0001. NS denotes not significant.

Both unstimulated and TNF-α-stimulated HUVECs and HCAECs were treated with either 0.05% DMSO vehicle control or 25 μM iCRT-14 for 18 h; endothelial cells were then co-cultured with activated platelets and adherence quantified. TNF-α did not significantly enhance platelet adhesion to HUVECs or HCAECs (Figures 5A, B). Endothelial-platelet interaction was unaffected by iCRT-14 in unstimulated cells; interestingly, levels of bound platelets were significantly increased by iCRT-14 in TNF-α-stimulated HUVECs and HCAECs (Figures 5A, B). To determine whether these findings translated to an *ex vivo* model, intact segments of human saphenous vein treated with either 0.05% DMSO or 25 μM iCRT-14 for 18 h, then co-cultured with activated platelets and bound platelets counted. As observed in isolated endothelial cells, treatment with iCRT-14 increased platelet adhesion (Figure 5D).

The endothelial vitronectin receptor (integrin α_*v*_β_3_) mediates platelet-endothelial interaction (27, 28). In TNF-α-stimulated HUVECs, Western blotting demonstrated up-regulation of integrin α_*v*_ compared to the untreated controls; this induction was significantly reversed by iCRT-14 (Figures 5C, E). Treatment with iCRT-14 did not alter integrin α_*v*_ protein levels in unstimulated HUVECs (Figures 5C, E). Neither activation with TNF-α nor treatment with iCRT-14 influenced integrin β_3_ expression in HUVECs (Figure 5C).

Ultra-large multimers of von Willebrand factor (vWF) presented at the endothelial surface are reported to facilitate interaction of platelets and platelet microparticles with the intact endothelium (29, 30). Specifically, the A1 domain of membrane-bound endothelial vWF binds platelet glycoprotein Ibα and thereby promotes platelet recruitment and aggregation (29, 30). Ultra-large vWF multimers are stored in the Weibel-Palade bodies of endothelial cells and are released upon endothelial cell activation (31, 32); here, storage of vWF in Weibel-Palade bodies was visualized by immunostaining permeabilised HUVECs for vWF. In unstimulated HUVECs, iCRT-14 did not exert any effect on the amount of vWF stored in Weibel-Palade bodies (Figures 5F, G). As anticipated, activation of HUVECs with TNF-α lowered Weibel-Palade body-associated vWF; this action was not amplified or otherwise influenced by addition of iCRT-14 (Figures 5F, G). To evaluate levels of surface-bound vWF, immunofluorescence for vWF was performed on non-permeabilised HUVECs: TNF-α stimulation did not change the amount of membrane-bound vWF with respect to control cells; levels of membrane-tethered vWF were however markedly elevated by iCRT-14 in TNF-α-activated HUVECs but not unstimulated HUVECs (Figures 5H, I).

Ultra-large vWF multimers associated with the endothelial surface are proteolytically cleaved at the site of the A2 domain by ADAMTS13 (A disintegrin-like and metalloprotease with thrombospondin type-1 repeats-13) such that the platelet-interacting A1 domain of vWF is released (33, 34). Western blotting performed on conditioned culture medium collected from HUVECs confirmed an elevation in levels of ADAMTS13 and soluble vWF following treatment with TNF-α (Figures 5C, J, K). Addition of iCRT-14 produced a significant decline in TNF-α-induced ADAMTS13 and soluble vWF protein levels; this response did not occur in unstimulated HUVECs (Figures 5C, J, K).

### Inhibition of Wnt/β-catenin signaling moderately impaired endothelial regrowth following wounding

**FIGURE 6:**
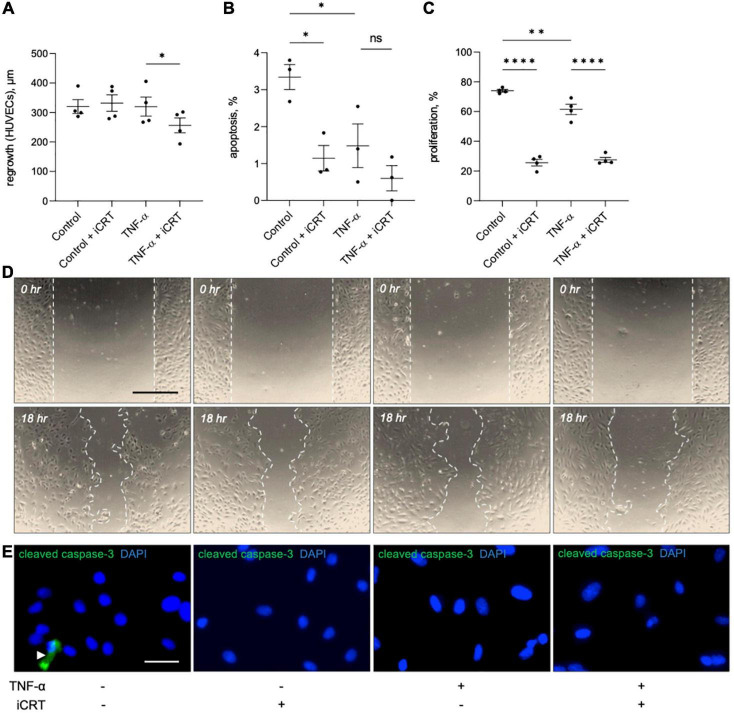
Effects of inhibition of Wnt/β-catenin signaling on wound healing, apoptosis and proliferation in cultured endothelial cells. Human umbilical vein endothelial cells (HUVECs) were treated with either 0.05% DMSO vehicle control or 25 μM inhibitor of β-catenin-responsive transcription (iCRT) in the presence or absence of 10 ng/mL recombinant human tumor necrosis factor-α (TNF-α) stimulus for 18 h. **(A)** HUVECs were subjected to scratch wounding and regrowth was quantified; data is expressed in μm (*n* = 4 each). **(B)** HUVECs were subjected to immunofluorescence for cleaved caspase-3, and apoptosis quantified and expressed as the percentage of cleaved caspase-3-positive cells (*n* = 3). **(C)** HUVECs were subjected to fluorescent labeling of incorporated EdU, and proliferation quantified and expressed as the percentage of EdU-positive cells (*n* = 4). **(D)** Representative images of scratch wound assay performed on HUVECs. Dashed line indicates wound edge. Scale bar represents 500 μm. **(E)** Representative images of HUVECs immunostained (green) for cleaved caspase-3. White arrowhead indicates cleaved caspase-3-positive cell. Nuclei were stained with DAPI (blue). Scale bar represents 10 μm and applies to all panels. *Indicates *p* < 0.05, ^**^p < 0.01, ^***^p < 0.001, and ^****^p < 0.0001. NS denotes not significant.

The effect of iCRT-14 on endothelial cell regrowth, survival and proliferation was examined to evaluate whether the compound would impair post-operative re-endothelialisation in saphenous vein grafts. Scratch wound assays indicated that iCRT-14 did not influence wound closure in unstimulated HUVECs but did moderately retard re-endothelialisation in TNF-α-stimulated cells (Figures 6A, D). Inhibition of β-catenin activity with iCRT-14 significantly enhanced cell survival in unstimulated HUVECs but had no effect on apoptosis in TNF-α-treated cells, as determined by immunofluorescence for cleaved caspase-3 (Figure 6B). Treatment with iCRT-14 suppressed cell replication in unstimulated and TNF-α-activated HUVECs, which was analyzed by quantifying EdU incorporation (Figure 6C).

## Discussion

Altered canonical Wnt/β-catenin and NFκB signaling have been associated with atherosclerosis and other inflammatory disorders. Reciprocal crosstalk between both pathways forge a complex regulatory network that, depending on context, may be inhibitory or stimulatory (13). In this study, in primary cultures of endothelial cells, we demonstrated that suppressing β-catenin activity with the pharmacological inhibitor iCRT-14 reduced levels of NFκB protein in both the nuclear compartment and in whole cell lysates. We further reported that treatment with iCRT-14 significantly diminished TNF-α-driven secretion of inflammatory cytokines IL-8 and MCP-1, but not IL-6. Hence, our findings indicate that in cultured endothelial cells stimulated with TNF-α, Wnt/β-catenin signaling has a stimulatory effect on NFκB activity and supports a pro-inflammatory phenotype. Interestingly, the majority of the current literature on this subject matter presents the Wnt/β-catenin pathway as anti-inflammatory and as an antagonist of NFκB activity (13, 35–40); however, some studies have reported positive modulation of NFκB signaling by β-catenin (41–46), as reported here. For example, in one such study, in cultured human bronchial epithelial cells, siRNA-mediated silencing of β-catenin impaired lipopolysaccharide (LPS)-induced NFκB activation and blunted LPS-driven up-regulation of inflammatory cytokines IL-6, IL-8, MCP-1, TNF-α, and interleukin 1β (IL-1β) (46). This and other studies highlight the importance of context in determining the nature of crosstalk between the Wnt/β-catenin and NFκB signaling pathways.

Inhibition of β-catenin activity with iCRT-14 appeared to impair TNF-α-driven nuclear translocation of NFκB, suggesting that iCRT-14 might have promoted IκBα-dependent cytoplasmic retention of NFκB. Numerous mechanisms described for positive regulation of NFκB activity by the Wnt/β-catenin signaling pathway involve enhanced degradation of IκBα; for example, β-catenin/TCF-mediated induction of β-TrCP expression (β-transducin repeats-containing protein) facilitates β-TrCP-mediated ubiquitination of IκBα and subsequent proteosomal degradation, thereby enabling NFκB activation (41, 47). However, treatment with iCRT-14 did not restore IκBα levels quenched by TNF-α stimulation, indicating that other IκBα-independent mechanisms are responsible for the reduction in the levels of nuclear NFκB.

Analysis of whole cell lysates showed that treatment of TNF-α-stimulated HUVECs with iCRT-14 significantly lowered total levels of NFκB protein, indicating that β-catenin inhibition may have influenced NFκB stability and/or synthesis. The observed depletion of nuclear NFκB may have hence been a consequence of an overall reduction in NFκB levels as opposed to an impairment of nuclear translocation of NFκB by iCRT-14. Peroxisome proliferator activated receptor-γ (PPAR-γ) reportedly mediates ubiquitination and proteasome-dependent degradation of both cytoplasmic and nuclear NFκB (48). In mammalian cells, PPAR-γ and β-catenin operate in an opposite manner (49–54). Furthermore, Liu et al. demonstrated that mutation of key residues within the LEF/TCF binding domain of β-catenin abolish its ability to interact with PPAR-γ and inhibit PPAR-γ activity (55). As mentioned, it is thought that iCRT-14 disrupts β-catenin transcriptional activity by directly blocking the interface *via* which β-catenin and TCF4 interact. Hence, it is conceivable that iCRT-14 also blocks β-catenin-PPAR-γ interaction, thereby promoting PPAR-γ activity and PPAR-γ-driven ubiquitination and degradation of NFκB. However, further investigation is needed to advance and solidify our understanding of mechanisms underlying positive regulation of NFκB activity by the Wnt/β-catenin signaling pathway.

We have provided evidence here that inhibition of β-catenin activity with iCRT-14 notably restricts monocyte adhesion to both venous and arterial endothelial cells activated by TNF-α; therefore, we present a potential strategy for alleviating immune cell infiltration into the vessel wall during atherosclerosis and vein graft remodeling. Regarding the mechanism of this effect, treatment with iCRT-14 attenuated TNF-α-driven expression of VCAM-1 protein, but not ICAM-1 protein, though NFκB-dependent transcriptional activation of both VCAM-1 and ICAM-1 is well-documented (16). The Wnt/β-catenin pathway appears to differentially regulate NFκB target genes (38, 56). In human lung fibroblasts stimulated with IL-1β, activation of Wnt/β-catenin signaling with Wnt-3a had divergent effects on NFκB target genes: VCAM-1 transcript levels were further elevated, while ICAM-1 expression was uninfluenced, and IL-6 gene expression was repressed (38). These data align with our observations in endothelial cells, and together our studies further expose the sophisticated and complex nature of crosstalk between the Wnt/β-catenin and NFκB signaling pathways.

Increased vascular permeability often underlies the development and progression of cardiovascular diseases (57). We demonstrate here that following TNF-α challenge, inhibition of Wnt/β-catenin with iCRT-14 partially recovers endothelial monolayer integrity in a fashion that appears to be independent of VE-cadherin and dependent on tight junctions. In addition to cell-cell adhesion, anchorage of the endothelial lining to the underlying matrix *via* focal adhesions is essential for maintenance of barrier function (58). Here, treatment of cultured endothelial cells with iCRT-14 augmented the number of phospho-paxillin (Tyr118)-positive, active focal adhesions. Current literature suggests that the effect of tyrosine 118 phosphorylation on vascular integrity depends on context, with most sources interestingly pointing to a resultant increase in endothelial permeability (59). Fu et al., however, demonstrated that in human lung microvascular endothelial cells, hepatocyte growth factor challenge enhanced endothelial barrier function and that this response required Y118 paxillin phosphorylation (60). Hence, in addition to restoring tight junctions, it is conceivable that iCRT-14 improved endothelial monolayer integrity *via* elevating the number of focal adhesions engaged with and anchored to the underlying extracellular matrix.

**FIGURE 7:**
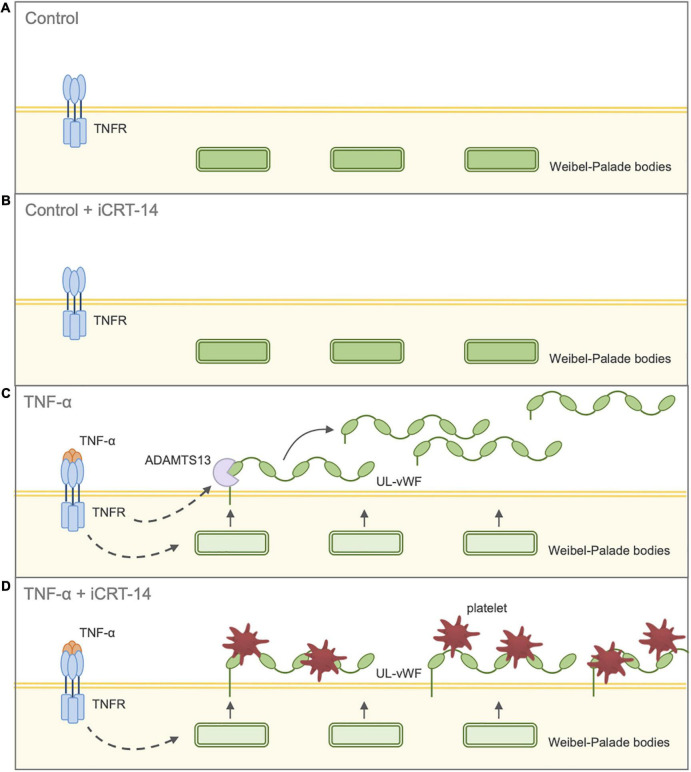
Schematic diagram illustrating effect of inhibition of Wnt/β-catenin signaling on endothelial-platelet interaction in unchallenged and TNF-α-challenged cultured endothelial cells. Endothelial cells were treated with either 0.05% DMSO vehicle control or 25 μM iCRT in the presence or absence of 10 ng/mL recombinant human TNF-α stimulus for 18 h, then co-cultured with thrombin-activated platelets for 10 min. **(A)** Control: Unchallenged endothelial cells treated with 0.05% DMSO vehicle control. UL-vWF multimers remain stored in the Weibel-Palade bodies. **(B)** Control + iCRT-14: Unchallenged endothelial cells treated with 25 μM iCRT-14. UL-vWF multimers remain stored in the Weibel-Palade bodies. **(C)** TNF-α: TNF-α-challenged endothelial cells treated with 0.05% DMSO vehicle control. TNF-α stimulates release of UL-vWF from Weibel–Palade bodies. TNF-α-driven up-regulation in ADAMTS13 results in proteolytic cleavage of membrane-tethered UL-vWF; hence, TNF-α-stimulation does not promote endothelial-platelet interaction. **(D)** TNF-α + iCRT-14: TNF-α-challenged endothelial cells treated with 25 μM iCRT-14. TNF-α stimulates release of UL-vWF from Weibel–Palade bodies. Treatment with iCRT-14 blocks TNF-a-mediated up-regulation of ADAMTS13 thereby maintaining high levels of membrane-tethered UL-vWF; hence, platelet recruitment is enhanced. Acronyms: ADAMTS13 - a disintegrin-like and metalloprotease with thrombospondin type-1 repeats-13; TNF-α - tumor necrosis factor-a; UL-vWF - ultra-large von Willebrand factor.

Our findings have so far suggested that inhibition of Wnt/β-catenin signaling holds potential therapeutic benefit by suppressing inflammatory responses and maintaining vascular integrity. The effect of iCRT-14 on platelet recruitment was then examined to evaluate whether the compound may adversely promote thrombus formation in either unstimulated or activated endothelial cells. Platelet adhesion assays revealed that treatment of both HUVECs and HCAECs with iCRT-14 facilitated endothelial-platelet interaction in TNF-α-challenged cells but not unchallenged cells. These findings were mirrored *ex vivo* in surgically prepared segments of human saphenous vein, where harvesting and handling of the venous material itself is known to damage and activate the endothelium (7–9). We propose that treatment with iCRT-14 facilitated platelet binding to TNF-α-activated endothelial cells by blocking the observed TNF-α-induced increase in secreted ADAMTS13 protein levels, thereby maintaining high levels of membrane-bound vWF (Figure 7). We suggest that treatment of unstimulated endothelial cells with iCRT-14 did not promote platelet adhesion as this requires TNF-α-driven release of vWF from Weibel-Palade bodies (Figure 7). Regarding the action of iCRT-14 in platelet recruitment, our data do not suggest a role for the vitronectin receptor. Of note, activation of cultured endothelial cells with TNF-α alone did not promote platelet binding; Tull et al. reported similar results but added that TNF-α did markedly enhance platelet adhesion when administered in combination with other inflammatory cytokines such as IL-1β and TGF-β1 (61). It may be that in endothelial cells stimulated with TNF-α alone, ADAMTS13-mediated proteolytic cleavage of membrane-tethered vWF hinders endothelial-platelet interaction. However, involvement of other inflammatory cytokines such as TGF-β1 alters this event such that a matrix of membrane-tethered vWF can form across the endothelial surface (61). Post-translational regulation of vWF is hence also sophisticated and complex and so the pro-coagulatory effects of Wnt/β-catenin inhibition highlighted here need further investigation.

In this study, we have revealed that inhibition of Wnt/β-catenin signaling in TNF-α-activated endothelial cells holds multiple anti-inflammatory benefits including a reduction in inflammatory cytokine production, a decline monocyte recruitment and recovery of vascular integrity. This was demonstrated in cultured endothelial cells of both venous and arterial origin, and therefore has relevance for vein graft failure and atherosclerosis, respectively. The observed pro-thrombotic effects of iCRT-14 treatment suggest that caution should be exercised in the employment of Wnt/β-catenin inhibitors as a therapy for vein graft stenosis and atherosclerosis; however, this effect may diminish in an alternative context, such as an *in vivo* environment, or when administered in conjunction with anti-platelet therapies such as aspirin and clopidogrel. Furthermore, some studies substantiate that in regions of endothelial denudation, platelet recruitment is in fact beneficial for re-endothelialisation (62, 63). The anti-proliferative action of iCRT also implies that it may not be a suitable therapy immediately following CABG surgery so as to allow re-endothelialisation of the vein graft; the pro-survival effect of Wnt/β-catenin inhibition may however be useful in the prevention of endothelial loss in late-stage atherosclerosis.

## Data availability statement

The original contributions presented in this study are included in the article/Supplementary material, further inquiries can be directed to the corresponding author.

## Ethics statement

The studies involving human participants were reviewed and approved by South West Frenchay Research Ethics Committee and NRES Committee East of England–Norfolk, The Old Chapel, Royal Standard Place, Nottingham, NG1 6FS. The patients/participants provided their written informed consent to participate in this study.

## Author contributions

KW performed the experiments presented in all figures and drafted the manuscript. AS performed the experiments presented in Table 1. GL and HB performed the experiments presented in Figure 3. JC performed the experiments presented in Figure 5. AF performed and/or oversaw experiments presented in Figures 3, 4 and Table 1. LH assisted with experimental design of experiments in Figure 5. AP oversaw and assisted with experimental design of experiments in Figure 5. JJ oversaw the project. SG secured funding, oversaw the project, and edited the manuscript. All authors contributed to the article and approved the submitted version.
