# Supplementary figures and images for: Pro-inflammatory role of Wnt/β-catenin signaling in endothelial dysfunction
Source: Front Cardiovasc Med. 2023 Jan 17;9:1059124. doi: 10.3389/fcvm.2022.1059124 (PMC9923234; doi:10.3389/fcvm.2022.1059124)

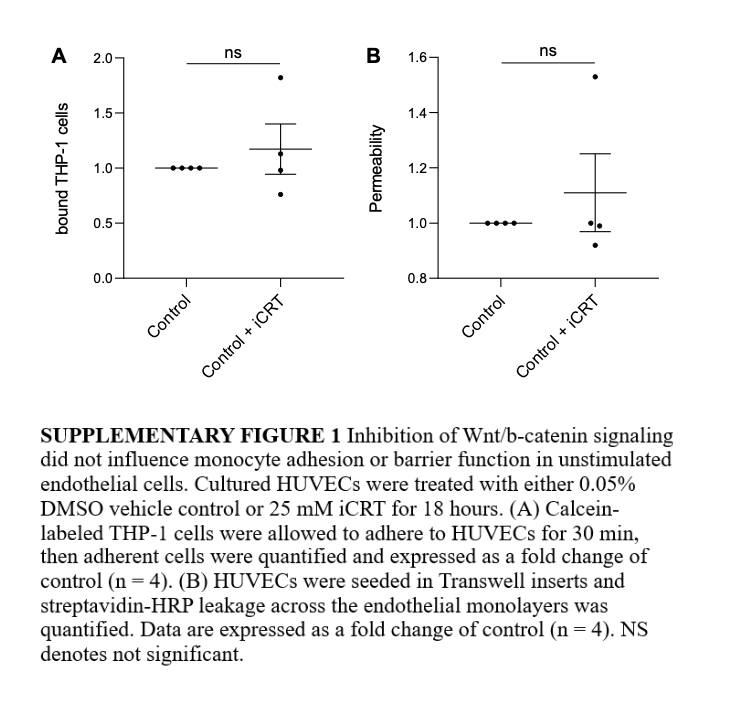

Supplement: Supplementary file 1 [file Image_1.tiff]
